# Sirtuin 7 Regulates Nitric Oxide Production and Apoptosis to Promote Mycobacterial Clearance in Macrophages

**DOI:** 10.3389/fimmu.2021.779235

**Published:** 2021-12-03

**Authors:** Su Zhang, Yaya Liu, Xuefeng Zhou, Min Ou, Guohui Xiao, Fang Li, Zhongyuan Wang, Zhaoqin Wang, Lei Liu, Guoliang Zhang

**Affiliations:** ^1^ National Clinical Research Center for Infectious Diseases, Shenzhen Third People’s Hospital, Southern University of Science and Technology, Shenzhen, China; ^2^ Department of Clinical Oncology, The University of Hong Kong-Shenzhen Hospital, Shenzhen, China; ^3^ School of Medical Technology, Guangdong Medical University, Dongguan, China

**Keywords:** *Mycobacterium tuberculosis*, SIRT7, nitric oxide, apoptosis, macrophages

## Abstract

The host immune system plays a pivotal role in the containment of *Mycobacterium tuberculosis* (Mtb) infection, and host-directed therapy (HDT) is emerging as an effective strategy to treat tuberculosis (TB), especially drug-resistant TB. Previous studies revealed that expression of sirtuin 7 (SIRT7), a nicotinamide adenine dinucleotide (NAD^+^)-dependent deacetylase, was downregulated in macrophages after Mycobacterial infection. Inhibition of SIRT7 with the pan-sirtuin family inhibitor nicotinamide (NAM), or by silencing SIRT7 expression, promoted intracellular growth of Mtb and restricted the generation of nitric oxide (NO). Addition of the exogenous NO donor SNAP abrogated the increased bacterial burden in NAM-treated or SIRT7-silenced macrophages. Furthermore, SIRT7-silenced macrophages displayed a lower frequency of early apoptotic cells after Mycobacterial infection, and this could be reversed by providing exogenous NO. Overall, this study clarified a SIRT7-mediated protective mechanism against Mycobacterial infection through regulation of NO production and apoptosis. SIRT7 therefore has potential to be exploited as a novel effective target for HDT of TB.

## Introduction

Tuberculosis (TB) is one of the top 10 causes of death worldwide. Globally in 2019, an estimated 8.9–11.0 million people fell ill with TB. Especially the drug-resistant tuberculosis is the most serious challenges in the clinical treatment of tuberculosis due to lack of effective drugs (1). Consequently, there is an urgent need for new strategies to control TB. Most individuals infected with *Mycobacterium tuberculosis* (Mtb) remain healthy, with less than 10% of individuals with latent TB eventually developing active TB (2). Thus, symptomatic tuberculosis is the result of the host immune system failing against Mtb. While Mtb has evolved diverse strategies to escape the host immune response, host-directed therapies (HDTs) have emerged as a promising area of research to fight TB (3).

Histone acetyltransferases (HATs) and histone deacetylases (HDACs) have central roles in regulating acetylation modification of both histone and non-histone substrates. Acetylation, an important post-translational modification that regulates gene expression, is involved in the regulation of cancer, cell stress, metabolism, aging, and infectious diseases (4). HDACs can be divided into four classes based on their protein structure and sequence homology. Sirtuins belong to the class III HDACs, which are NAD^+^-dependent enzymes with protein deacetylase and ADP-ribosylase activities, and comprise the members SIRT1 to SIRT7 (5). SIRT7—the least-studied sirtuin to date—is capable of deacetylation activity (6–8) and has also been shown to exhibit desuccinylation (9), deacylase (10), depropionylation (11), and deglutarylation activities (12).

Nitric oxide (NO) is instrumental in the pathogenesis of TB. NO and ${\text{O}}_{2}^{-}$ combine to form highly Reactive Nitrogen Species (RNS) such as $\text{N}{\text{O}}_{3}^{-}\text{ and N}{\text{O}}_{2}^{-}$ within infected macrophages to drive bacterial death (13). Besides the direct activity against Mtb, NO also mediate apoptosis of activated macrophages to kill intracellular Mtb (14). NO regulating apoptosis appears to be specific to the type of cells, NO activates apoptosis in many cell types including macrophages, pancreatic islets, neurons, and thymocytes (15).

In this study, the role of SIRT7 in the pathogenesis of TB was deciphered using a pan-sirtuin family inhibitor NAM and SIRT7-knockdown macrophages. Mycobacterial infection led to downregulation of SIRT7, and inhibition of SIRT7 activity by nicotinamide (NAM) or knockdown of SIRT7 expression increased the risk of Mycobacterial infection. Concurrently, inhibition of SIRT7 activity by NAM or knockdown of SIRT7 expression also suppressed NO release and apoptosis of macrophages after Mycobacterial infection. Furthermore, this effect of SIRT7 inhibition could be alleviated by addition of the NO donor SNAP, and the level of apoptosis was also enhanced by adding SNAP. In conclusion, this study revealed a novel anti-TB mechanism, with SIRT7 restricting both intracellular Mycobacteria growth by NO release and NO-dependent apoptosis. These findings indicate that SIRT7 could be exploited as a HDT against Mycobacterial infection.

## Results

### Mycobacterial Infection Downregulates SIRT7 Expression

**Figure 1:**
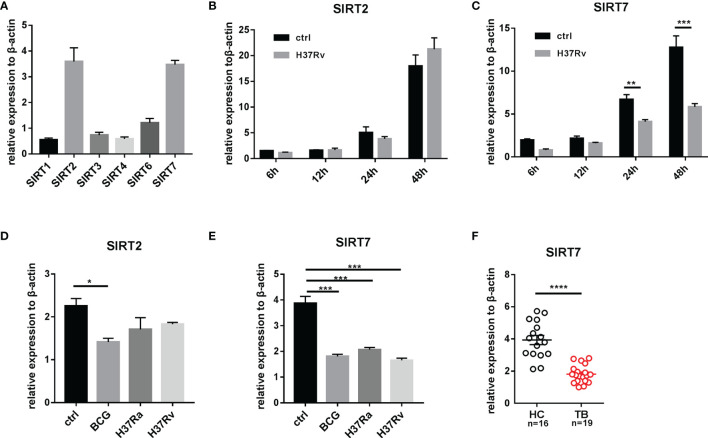
Mycobacteria infection downregulates SIRT7 expression as determined by quantitative RT-PCR analysis. **(A)** Expression levels of sirtuin family members in Raw264.7 cells. **(B, C)** SIRT2 and SIRT7 mRNA expression in Raw264.7 cells at 6, 12, 24, and 48 h after infection with H37Rv (MOI 10:1). **(D, E)** SIRT2 and SIRT7 mRNA expression in Raw264.7 cells infected with Mycobacterial strains with differing virulence. Cells were infected with BCG, H37Ra, and H37Rv, respectively (MOI 10:1) for 4 h, then SIRT2 and SIRT7 expression levels were analyzed 24 h after infection. **(F)** Differences in expression of SIRT7 in hMDMs from healthy individuals and patients with TB. Data are representative of three independent experiments with similar results and are presented as means ± SD. Two way ANOVA was performed in **(B, C)**, One way ANOVA was performed in **(D, E)**, Unpaired Student’s t-test was used in **(F)**. *p < 0.05; **p < 0.01; ***p < 0.001; ****p< 0.0001.

The sirtuins (SIRTs) family comprises seven members (SIRT1–7) that are dispersed amongst different cellular compartments. The expression level of each member of the SIRT family was detected in Raw264.7 cells by RT-PCR. SIRT2 and SIRT7 exhibited the highest levels of expression (**Figure 1A**), thus further experiments focused on the role of SIRT2 and SIRT7 in Mycobacterial infection. A time-dependent reduction in SIRT7 mRNA expression was observed in Raw264.7 cells infected with the Mtb virulent strain H37Rv, while there were no significant changes in SIRT2 mRNA expression before and after infection (**Figures 1B, C**). Raw264.7 cells were subsequently infected with virulent Mtb H37Rv, avirulent Mtb H37Ra or M. bovis BCG for 24 h, and no significant differences in SIRT2 and SIRT7 expression levels were detected between the Mycobacteria of differing virulence (**Figures 1D, E**). Similar results were detected in human cell lines THP1 (**Supplementary Figures 1A, B**). Furthermore, human monocyte-derived macrophages (hMDMs) obtained from peripheral blood mononuclear cells (PBMCs) of patients with active TB also displayed reduced SIRT7 mRNA expression relative to healthy controls (**Figure 1F**, The information of the patients and healthy controls was shown in **Table 1**). In summary, Mycobacterial infection downregulates SIRT7 expression, indicating an important disease-related effect of SIRT7 on Mtb infection.

**Table 1:** Characteristics of patients with active TB and healthy controls.

	Healthy	Active TB	*P*-value
Sample size (no.)	16	19	–
Age (years) (mean ± SD)	44.5 ± 16.22	51.32 ± 19.2	0.2700
Sex (M/F)	10/6	14/5	0.4777

F, Female; M, male. The level of significance was evaluated by unpaired student t-test or Chi-square test. P-value < 0.05 was considered statistically significant.

### SIRT7 Inhibitor NAM Increases the Mycobacteria Burden in Macrophages

**Figure 2:**
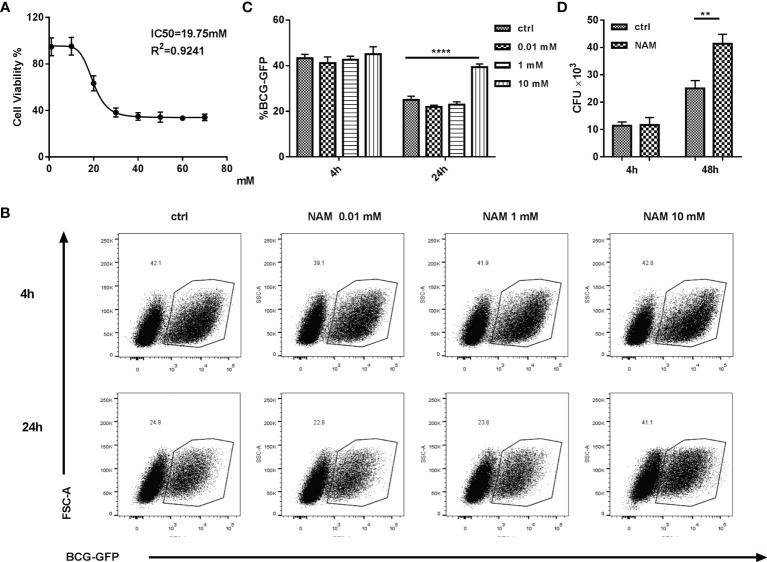
SIRT7 inhibitor NAM increases Mycobacteria burden in Raw264.7 cells. **(A)** Dose-dependent cytotoxicity and IC_50_ values of NAM in Raw264.7 cells. **(B, C)** Raw264.7 cells were pretreated with the indicated doses of NAM 24 h before infection with BCG-GFP (MOI 10:1), and then analyzed by flow cytometry at 4h and 24 h after infection. Representative flow cytometry images of BCG-GFP-positive Raw264.7 cells were captured **(B)** and the percentage of cells positive for GFP were calculated using Flow Jo software **(C)**. **(D)** Colony-forming unit (CFU) counts from Raw264.7 cells treated with or without NAM (10 mM) after 4h and 48h infected with H37Rv. Data represent means ± SD for three independent experiments. Two way ANOVA was performed in **(C, D).** **p < 0.01; ****p< 0.0001.

To determine whether SIRT7 was essential for controlling Mycobacteria growth, the SIRT7 inhibitor NAM was used to treat Mycobacteria-infected cells. NAM non-selectively inhibits the sirtuin family through competition binding to the NAD+ binding site of the sirtuins (16). The safety of the NAM has been verified and the IC_50_ value was 19.75 mM in Raw264.7 cells (**Figure 2A**). To evaluate whether NAM affected the Mycobacteria burden in macrophages, Raw264.7 cells were pretreated and maintained with 0.01, 1, or 10 mM NAM for 24 h and then infected with a BCG fluorescent reporter strain that expresses GFP (BCG-GFP, MOI 10:1) and analyzed by flow cytometry at 4h, 24h respectively. The results showed that NAM had no significant impact on the phagocytosis rate, but NAM at a concentration of 10 mM increased the Mycobacteria burden in Raw264.7 cells, while lower concentrations of NAM (0.01 and 1 mM) had no effect on the Mycobacteria burden of the cells as indicated by the percentage of GFP-positive cells (**Figures 2B, C**). The number of colony-forming units (CFUs) from lysed Raw264.7 cells infected with H37Rv were also tested at 4h, 48h after infection. The results were consistent with the experiment using BCG-GFP (**Figure 2D**). These findings suggest that SIRT7 restricts intracellular Mycobacteria growth and can be inhibited by the non-selective inhibitor NAM.

### SIRT7 Knockdown Increases the Mycobacteria Burden While Overexpression of SIRT7 Protects Cells From Mycobacteria

**Figure 3:**
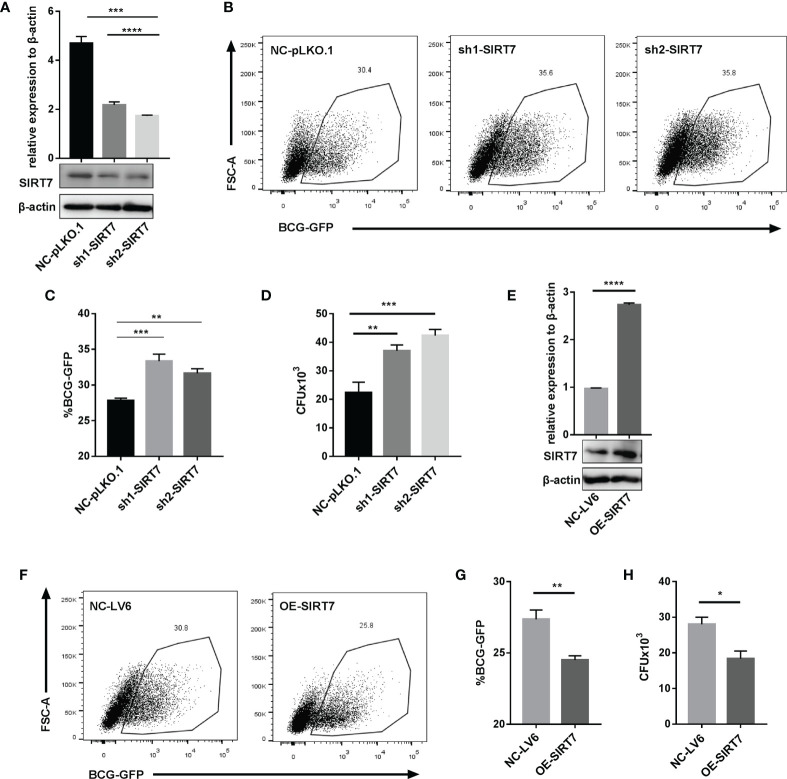
SIRT7 knockdown increases the risk of Mycobacteria infection, while overexpression of SIRT7 protects cells from Mycobacteria. **(A–D)** Raw264.7 cells stably expressing scrambled control (NC-pLKO.1) and two independent SIRT7 shRNAs (sh1-SIRT7 and sh2-SIRT7), respectively, were established. SIRT7 expression levels in these cells were measured by quantitative RT-PCR and Western blot analysis **(A)**. Representative flow cytometry images **(B)** and percentage **(C)** of GFP-positive cells were recorded in the scrambled control and SIRT7-knockdown cells 24 h after infection with BCG-GFP (MOI 10:1). **(D)** Colony-forming unit (CFU) counts in scrambled control and SIRT7-knockdown cells after 48h infection with H37Rv. **(E–H)** Raw264.7 cells stably overexpressing SIRT7 (OE-SIRT7) and vector control (NC-LV6) were established. SIRT7 expression levels in these cells were measured by quantitative RT-PCR and Western blot analysis **(E)**. Representative flow cytometry images **(F)** and percentage **(G)** of GFP-positive cells were recorded in control and SIRT7-overexpressing cells 24 h after infection with BCG-GFP (MOI 10:1). **(H)** CFU counts in vector control and SIRT7-overexpressing cells after 48h infected with H37Rv. Data are representative of three independent experiments with similar results and are presented as means ± SD. One way ANOVA was performed in **(A, C, D)**, Unpaired Student’s t-test was used in **(E, G, H)**. *p < 0.05; **p < 0.01; ***p < 0.001; ****p< 0.0001.

To further explore whether SIRT7 was essential for controlling mycobacterial growth, SIRT7-deficient Raw264.7 cells were constructed. Lentivirus particles containing SIRT7 shRNA and scrambled control were transduced into Raw264.7 cells, and SIRT7 expression was determined by quantitative RT-PCR and Western blotting after filtering and selecting stably expression cells using puromycin (4 µg/ml) (**Figure 3A**). Flow cytometry analysis of BCG-GFP-infected cells 24h after infection and CFU counts of H37Rv-infected cells 48h after infection revealed that SIRT7 knockdown macrophages had higher bacillary loads compared with those of the control cells (**Figures 3B** **–D**). But there was no significant differences at 4h after infection between the control and SIRT7 knockdown cells (**Supplementary Figures 2A–C**). In addition, SIRT7 and vector were transduced into Raw264.7 cells by lentivirus (**Figure 3E**). As expected, overexpression of SIRT7 inhibited the growth of intracellular Mycobacteria as indicated by flow cytometry analysis and CFU counts (**Figures 3F** **–H**) but had no significant impact on the phagocytosis rate (**Supplementary Figures 2D–F**). Together, these data indicate that SIRT7 contributes to the control of Mycobacteria growth in Raw264.7 cells.

### SIRT7 Regulates the Generation of NO in Mycobacteria-Infected Macrophages

**Figure 4:**
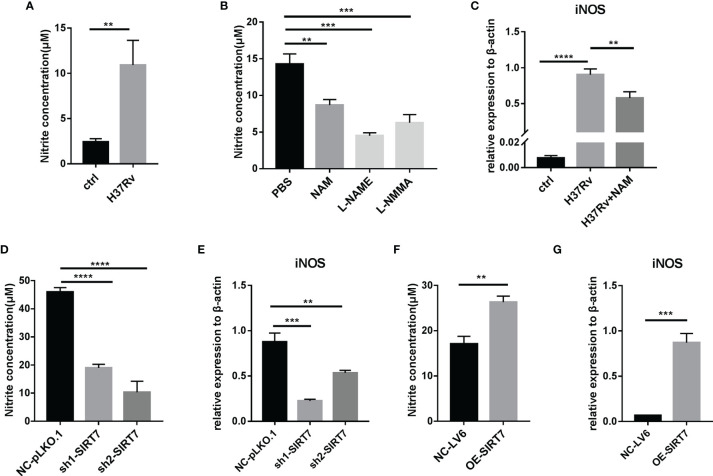
SIRT7 inhibitor NAM or SIRT7-knockdown inhibits nitric oxide (NO) production in Raw264.7 cells. **(A, B)** NO production in Raw264.7 cells was measured by Griess reaction assay 24 h after infection with H37Rv (MOI 10:1) **(A)** or after pretreatment of cells with NAM (10 mM) or NOS inhibitors L-NMME (500 μM) and L-NMMA (500 μM) **(B)**. **(C)** Quantitative RT-PCR analysis of *iNOS* expression levels in infected Raw264.7 cells pretreated with or without NAM. mRNA was collected from the cells 24 h after infection with H37Rv. **(D, E)** NO concentration **(D)** and quantitative RT-PCR analysis of *iNOS* **(E)** expression levels in Raw264.7 cells stably expressing scrambled control (NC-pLKO.1) or two independent SIRT7 shRNAs (sh1-SIRT7 and sh2-SIRT7). **(F, G)** NO concentration **(F)** and quantitative RT-PCR analysis of *iNOS* **(G)** in Raw264.7 cells stably overexpressing SIRT7 (OE-SIRT7) and vector control (NC-LV6). Data are representative of three independent experiments with similar results and are presented as means ± SD. One way ANOVA was performed in **(B–E)**, Unpaired Student’s t-test was used in **(A, F, G)**. **p < 0.01; ***p < 0.001; ****p< 0.0001.

NO is a key anti-mycobacterial molecule, and production of NO was increased in H37Rv-infected Raw264.7 cells (**Figure 4A**), consistent with reports in the literature (17). To explore whether SIRT7 had a role in NO production, the function of SIRT7 in Raw264.7 cells was inhibited by NAM (10 mM) and then the cells were infected with H37Rv for 24 h and the NO level was measured by Griess reagent method. Addition of NAM downregulated NO production in Raw264.6 cells, similar to the effect of NO synthase inhibitors L-NAME and L-NMMA (**Figure 4B**). The expression level of the NO synthase gene *iNOS* was also downregulated by NAM (**Figure 4C**). Further experiments were conducted to determine whether SIRT7 knockdown could inhibit NO production, and these experiments revealed that NO production after H37Rv infection was significantly decreased by SIRT7 knockdown (**Figure 4D**). The NO production-related gene *iNOS* which metabolize arginine to NO and citrulline was also downregulated by SIRT7 knockdown (**Figure 4E**). Moreover, overexpression of SIRT7 increased the generation of NO (**Figure 4F**) and the expression of iNOS (**Figure 4G**) in Raw26.47 cells. Furthermore, we detected the *Arg-1* which hydrolyzes arginine to ornithine and urea expression levels. The *Arg-1* expression could also be downregulated by SIRT7 knockdown an upregulated by SIRT7 overexprssion (**Supplementary Figures 3A, B**). However, the enhancement in iNOS levels was around one thousand times higher compared to that of Arg-1 in Mycobacteria-infected Raw264.7 cells (**Supplementary Figure 2C**), so we considered iNOS to play the major role in NO production modulation in Mycobacteria-infected Raw cells. Additionally, it was also reported that high Arg-1 expression preceded the increased induction of iNOS at early time points of infection with mycobacteria (18). These findings confirm that SIRT7-mediated inhibition of Mycobacteria in macrophages is NO dependent.

### The NO Donor SNAP Abrogates the NAM or SIRT7 Knockdown-Induced Increase Mycobacteria Burden in Macrophages

**Figure 5:**
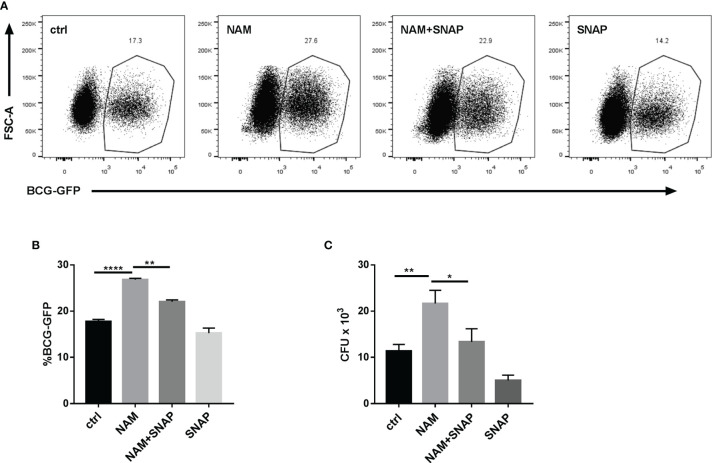
Nitric oxide (NO) donor SNAP abrogates the NAM-induced increase in Mycobacteria burden in Raw264.7 cells. **(A, B)** Raw264.7 cells were pretreated with NAM (10 mM) and/or SNAP (200 μM) 24 h before infection with BCG-GFP (MOI 10:1) and were then analyzed by flow cytometry 24 h after infection. Representative flow cytometry images **(A)** were recorded and the percentage of BCG-GFP-positive Raw264.7 cells **(B)** were calculated by Flow Jo software. **(C)** Colony-forming unit (CFU) counts from Raw264.7 cells treated with NAM (10 mM) and/or SNAP (200 μM) 48h after infection. Data represent means ± SD for three independent experiments. One way ANOVA was performed in **(B, C)**. *p < 0.05; **p < 0.01; ****p< 0.0001.

**Figure 6:**
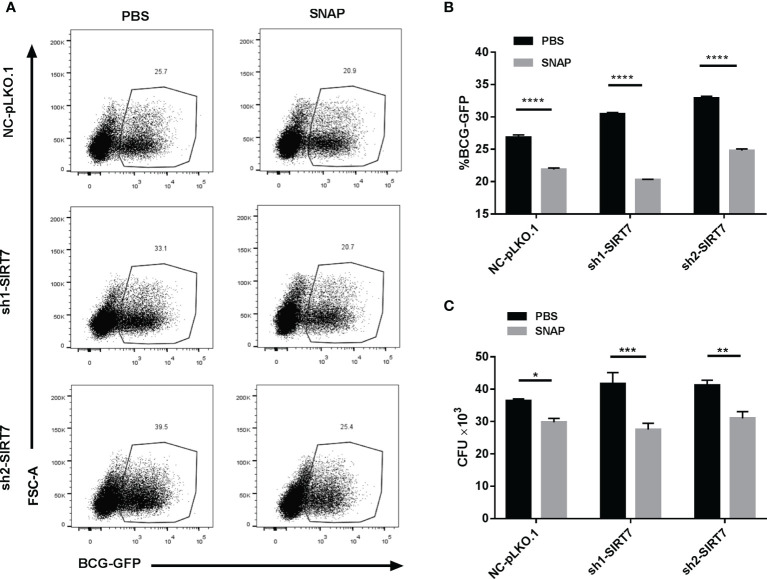
Nitric oxide donor SNAP abrogates the SIRT7-knockdown-induced increase in Mycobacteria burden in Raw264.7 cells. **(A, B)** SIRT7-knockdown Raw264.7 cells were pretreated with SNAP (200 μM) 24 h before infection with BCG-GFP (MOI 10:1) and were then analyzed by flow cytometry 24 h after infection. Representative flow cytometry images **(A)** were recorded and the percentage of BCG-GFP positive Raw264.7 cells **(B)** were calculated by Flow Jo software. **(C)** Colony-forming unit (CFU) counts of SIRT7-knockdown Raw264.7 cells treated with or without SNAP (200 μM) 48h after infection. Data represent means ± SD for three independent experiments. Two way ANOVA was performed in **(B, C)**. *p < 0.05; **p < 0.01; ***p < 0.001; ****p< 0.0001.

To confirm whether SIRT7 controlled Mycobacteria growth *via* NO, the NO donor SNAP (200 μM) was added to suppress the increase in bacterial load caused by NAM (10 mM), which inhibits the function of SIRT7. Compared with the group treated with NAM, flow cytometry analysis of BCG-GFP-infected cells and CFU counts of H37Rv-infected cells both revealed that SNAP alleviated the NAM-induced increase of Mycobacteria burden in Raw264.7 cells (**Figures 5A** **–C**). Correspondingly, SNAP addition also alleviated the SIRT7 knockdown-induced increase of Mycobacteria burden in Raw264.7 cells (**Figures 6A** **–C**). Earlier experiments in the current study suggested that SIRT7 regulated NO production in Mycobacteria-infected macrophages, and these findings confirm that SIRT7 restricts intracellular Mycobacteria growth by promoting NO release.

### SIRT7 Promoted Elimination of Intracellular Mycobacteria by NO-Dependent Apoptosis

**Figure 7:**
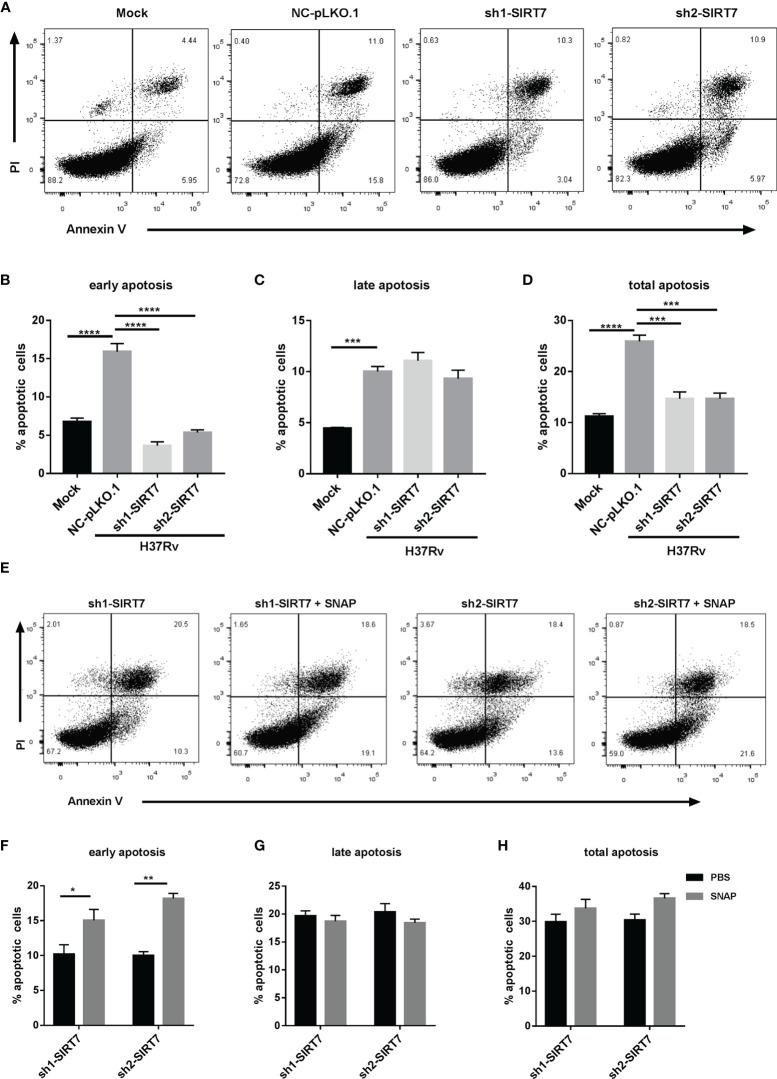
SIRT7 promotes elimination of intracellular Mycobacteria by nitric oxide-dependent apoptosis. Scrambled control (NC-pLKO.1) and SIRT7-knockdown (sh1-SIRT7 and sh2-SIRT7) Raw264.7 cells were infected with H37Rv and apoptosis was detected by Annexin V and propidium iodide (PI) staining. **(A, E)** Representative flow cytometry images of apoptotic cells. **(B–D)** Percentage of apoptotic cells (early, late, and total, respectively) in the scrambled control and SIRT7-knockdown cells. **(F–H)** Percentage of apoptotic cells (early, late, and total, respectively) in the SIRT7-knockdown cells pretreated with or without SNAP (200 μM). The percentages of early, late, and total apoptotic cells were calculated by Flow Jo software. Data are representative of three independent experiments with similar results and are presented as means ± SD. Two way ANOVA was performed in **(B–D)**, One way ANOVA was performed in **(F–H)**. *p < 0.05; **p < 0.01; ***p < 0.001; ****p< 0.0001.

Given that NO was reported to enhance apoptosis in macrophages (19), and apoptosis is usually considered to play a vital role in the host defense against Mtb (20), the ability of SIRT7 to regulate apoptosis in Mycobacteria-infected macrophages was evaluated. Following infection of cells with H37Rv for 24 h, the percentage of apoptotic cells was assessed by Annexin V/propidium iodide (PI) staining assay. Compared with the control group (Raw264.7 cells with scrambled control NC-pLKO.1), SIRT7-knockdown cells had markedly lower percentages of apoptotic cells in total (AnnexinV+) and early (AnnexinV+/PI–) apoptotic ratios, but there were no significant changes in late apoptotic ratios (**Figures 7A** **–D**). These findings suggested that SIRT7 promotes elimination of intracellular Mycobacteria by inducing apoptosis in macrophages. Earlier experiments in the study showed that SIRT7 promoted NO generation and increased the apoptosis ratio in macrophages, therefore an experiment was conducted to test whether NO played a role in the apoptosis of macrophages induced by SIRT7. SIRT7-knockdown Raw264.7 cells were treated with the NO donor SNAP (200 μM) and then infected with Mtb strain H37Rv. Addition of the NO donor led to significant increases in early apoptotic ratios, but there were no significant changes in late and total apoptotic ratios (**Figures 7E** **–H**). These findings confirm that SIRT7 promotes elimination of intracellular Mycobacteria by NO-dependent apoptosis.

## Discussion

Since Mtb has evolved diverse strategies to escape immune surveillance, HDTs that enhance Mtb-specific immunity are urgently needed. Sirtuins are a conserved family of proteins (seven recognized members to date) that are NAD+-dependent deacetylases (21). There are previous reports describing anti-Mtb activity of sirtuins. For example, activation of SIRT1 reduced intracellular growth of drug-susceptible and drug-resistant strains of Mtb and induced phagosome-lysosome fusion and autophagy in a SIRT1-dependent manner (22). SIRT1 suppressed TAK1 activation and the subsequent production of inflammatory cytokines *via* the MAPK and NF-κB pathways to fine-tune the excessive inflammatory response to Mtb infection (23). Furthermore, SIRT3 played an anti-Mtb role through coordinating mitochondrial and autophagic cell death functions (24–26), while SIRT2 was reported to play the opposite role in anti-Mtb activities. The SIRT2 inhibitor AGK2 reduced the bacillary load of both drug-sensitive and drug-resistant strains of Mtb (27). However, there remains a different view due to the absence of SIRT2 in myeloid cells not impacting lung cellular responses to Mtb (28). Furthermore, SIRT5 deficiency did not worsen endotoxemia, pneumonia caused by *Klebsiella pneumoniae* or *Streptococcus pneumoniae*, *Escherichia coli*-induced peritonitis, listeriosis, and staphylococcal infection (29). Therefore, different members of the sirtuin family have different roles in fighting Mtb infections. In the current study, the role of SIRT7 in Mtb infection was investigated for the first time. SIRT2 and SIRT7 were the members of the sirtuin family with dominant expression in macrophages. Mtb infection suppressed SIRT7 expression, but there were no significant changes in expression of SIRT2 after Mtb infection, congruent with results reported by Smulan et al. (28). Inhibition of SIRT7 activity by NAM or knockdown of SIRT7 expression increased the risk of Mtb infection. In addition, overexpression of SIRT7 inhibited the growth of intracellular Mtb.

The molecular mechanism of SIRT7 in the host immune response has been explored in recent years. SIRT7 deacetylated and promoted SMAD4 degradation to antagonize TGF-β signaling (30). TGF-β1 exhibited immunosuppressive activity and accelerated the progression of pulmonary TB (31). Loss of SIRT7 decreased expression of TNF, which was essential for protection against Mtb (32, 33). Furthermore, SIRT7 also suppressed the NF-κB signaling pathway to attenuate excessive inflammatory responses (34). In the current study, a new mechanism of SIRT7 anti-Mtb activity was discovered. SIRT7 restricted intracellular Mtb growth by promoting NO release from macrophages. The pan-sirtuin family inhibitor NAM and SIRT7 knockdown both downregulated the NO concentration and the expression level of NO release-related genes in macrophages. In contrast, overexpression of SIRT7 increased the generation of NO in macrophages. Furthermore, the NO donor SNAP abrogated the NAM- or SIRT7-knockdown-induced increase in Mtb burden in macrophages. These data further supported that SIRT7 restricts intracellular Mtb growth by promoting NO release.

In addition to its function as a reactive free radical to execute direct anti-Mtb activities (35), NO is also involved in innate immunity by inducing macrophage apoptosis (36). Thus, the mechanism of apoptosis regulation by SIRT7 was further explored. SIRT7 knockdown in macrophages suppressed early apoptosis of Mtb-infected Raw264.7 cells, while the NO donor SNAP promoted early apoptosis ratios after Mtb infection.

Overall, this study demonstrated for the first time, that SIRT7 has a crucial role in Mtb infection, and aside from regulating NO release to directly kill Mtb, SIRT7 also promoted elimination of intracellular Mtb by NO-dependent apoptosis. However, the molecular mechanism underlying SIRT7 regulation of NO release has yet to be elucidated, and the candidate substrate which SIRT7 directly deacetylates in TB pathogenesis remains unclear. These are the scientific questions that will be addressed in our future work. The findings from the current study suggest there is potential to target SIRT7 in the development of innovative HDTs to improve TB treatment outcomes.

## Materials And Methods

### Cell Culture

Mouse macrophage Raw264.7 cells were purchased from National Collection of Authenticated Cell Cultures, China, and maintained in Dulbecco’s modified Eagle’s medium (DMEM, Gibco, 11965-092) supplemented with 10% fetal bovine serum (FBS, Gibco, 10091148) and 1% penicillin- streptomycin (Gibco, 15140122). Cells were cultured in a humidified incubator at 37°C and 5% CO_2_. In infection experiments, no antibiotic was used.

### Bacteria Culture

M. bovis BCG-green fluorescent protein (BCG-GFP) and Mtb standard strains H37Rv, H37Ra, BCG were grown in Middlebrook 7H9 broth (BD Biosciences, 271310) supplemented with 10% Oleic Acid-Dextrose-Catalase (OADC) (BD Biosciences, 212240), 0.5% glycerol, and 0.05% Tween 80 at 37°C for 5 to 7 days to achieve mid-logarithmic phase (optical density at 600 nm [OD_600_] = 0.3 to 0.8), Cultures were harvested, resuspended in PBS with 0.05% Tween 20, 25% glycerol, and stored at -80°C. One vial of the stock was thawed to calculate CFU per milliliter. On the day of infection, mycobacteria were thawed, washed, and sonicated before use.

### Preparation of hMDM

This study was approved by the Ethics Committee of Shenzhen Third People’s Hospital (approval number: 2019-038). Informed written consents were obtained from participants prior to venous blood collection. Human peripheral blood mononuclear cells (PBMCs) were isolated by Ficoll density gradient centrifugation (Axis-Shield, AS1114547) and differentiated at 1x10^6^ cells/ml in complete 1640 culture medium supplemented with 30 ng/ml human M-CSF (Gibco, PHC9501) for approximately 7 days.

### Drug Administration

Raw264.7 cells were pre-treated with nicotinamide (NAM) (Sigma, 72340) 10mM, or SNAP (MedChemExpress, HY-121526) 200μM, or L-NAME (MedChemExpress, HY-18729A) 500 μM and L-NMMA (MedChemExpress, HY-18732A) 500 μM for 24h before Mtb infection.

### Cell Viability Assay

The Raw264.7 cells were seeded at 5x10^4^ cells/well in a 96-well plate in complete DMEM. Different concentration of NAM were added and cultured for an additional 24 h, CCK-8 reagent (Vazyme, A311-02) was added into the well (10 μl/well) and incubated at 37°C for 2 h to measure cell viability. The absorbance was detected at 450nm with a Varioskan LUX Multimode Microplate Reader(Thermo Fisher, Varioskan LUX Multimode Microplate Reader). The cell viability rate (%) of three independent experiments was calculated as the follows:


$$\text{Cell viability rate }(\%)=\frac{\text{OD of treated cells}-\text{OD of background}}{\text{OD of control cells}-\text{OD of background}}\times 100\%$$

IC50 values were calculated using a four-parameter logistic curve (GraphPad Prism 7.0).

### Lentiviral Vector Construction and Lentivirus Packaging

Targeted sequences - homologous to SIRT7 (GenBank: 209011) and scrambled sequences which had no homology with the mouse gene—were synthesized, annealed, and cloned in the lentiviral expression vector pLKO.1-TRC (Addgene: 10878). SIRT7 whole length CDS was cloned in the lentiviral expression vector pLVML-3×HA-MCS-IRES-Puro using Homologous Recombination strategy (Vazyme, C115), The primers were synthesized in Sangon Biotech (Guangzhou, China) and showed in **Supplementary Table 1**. HEK293T cells were co-transfected with the lentiviral expression vector, packaging vector psPAX2 and envelope vector pMD2.G using Lipofectamine 3000 (Invitrogen, L3000015). Culture supernatants were harvested at 48h, filtered with a 0.45-µm pore size filter.

### Raw264.7 Stable Cell Line Construction

Raw264.7 cells were infected with viral supernatants collected from HEK293T cells transfected with lentiviral constructs for 24h, Then washing three times with prewarmed sterile phosphate-buffered saline (PBS) to remove extracellular lentivirus. After 72 h, stable cell lines were sorted by puromycin(2 μg/ml), and the efficiency of knockdown or overexpress was determined by Western blotting.

### Mtb Infection and Enumeration of Colony Forming Units (CFU)

Raw264.7 cells were seeded at 2x10^5^ cells/well in a 24-well plate in complete DMEM and infected with Mtb strains H37Rv at a multiplicity of infection (MOI) of 10 for 4h. Then washing three times with prewarmed sterile PBS to remove extracellular bacteria, and cultured with complete DMEM at 37°C and 5% CO_2._ After 4h and 48h, Cells were lysed with PBS containing 0.1% SDS, and the lysates were gradient diluted on Middlebrook 7H10 agar (BD Biosciences, 262710) supplemented with 10% OADC, 0.5% glycerol plates and incubated vertically at 37°C for 2-3 weeks. Bacterial colonies were counted and colony-forming unit (CFU) were estimated as per dilution.

### BCG-GFP Infected Cells Analysis by Flow Cytometry

Raw264.7 cells were infected with BCG-GFP at a multiplicity of infection (MOI) of 10 for 4h. Then washing three times with prewarmed sterile PBS to remove extracellular bacteria, and cultured with complete DMEM at 37°C and 5% CO_2._ Cells were collected in FACS tubes and the percentages of GFP positive cells were measured by flow cytometry (BD Biosciences, Canton II) after 4h and 24h infection and analyzed by FlowJo X 10.0.7 according to the manufacturer’s protocol.

### Nitric Oxide Assay

The levels of NO were measured by commercial kits (Beyotime, S0021S) according to the manufacturers’protocols. Mtb infection method as was mentioned above, the supernatants were collected for detection after 48h infection. Griess Reagent I 50μl and Griess Reagent II 50μl were added to 50μl supernatants in order. Nitrite concentration was determined by spectrophotometry (540 nm) from a standard curve (0-100 mmol/L) derived from NaNO2.

### Apoptosis Assay

The apoptotic cells were measured by FACS using FITC Annexin V Apoptosis Detection Kit (BD Biosciences, 556547). Mtb infection method as was mentioned above, cells were washed twice with cold PBS and stained with the Annexin V- PI reagent. Cellular apotosis levels were detected by flow cytometry (BD Biosciences, Canton II) and analyzed by FlowJo X 10.0.7 according to the manufacturer’s protocol.

### Quantitative RT-PCR

Total RNA was isolated from cells using Total RNA Kit I (OMEGA, R6834-02) according to the manufacturer’s instructions. cDNA was synthesized using ClonExpress Ultra One Step Cloning Kit (Vazyme, C115-02) followed by qRT-PCR using SYBR Green HiScript II Q RT SuperMix for qPCR Kit (Vazyme, R223-01). Real-time quantitative RT-PCR analysis was performed using ABI ViiA7 Real-Time thermal cycler (Thermo Fisher, ABI). The primers used in the study were synthesized in Sangon Biotech (Guangzhou, China) and showed in **Supplementary Table 2**. The mRNA expression levels were normalized to β-actin, and fold induction was calculated by the ΔΔCT method. RT-qPCR was performed in triplicate.

### Western Blot

Cells were harvested and lysed in RIPA lysis buffer (Beyotime, P0013B) for 5 min on ice.The protein concentration of the resultant lysates was measured with a bicinchoninic acid (BCA) protein kit (Beyotime, P0010S). Equal amounts of protein from each sample were separated by SDS–PAGE and electro-blotted onto PVDF membranes. The membrane was blocked with 5% skim milk powder solution in PBS with Tween 20 (PBST) for 2h at room temperature and incubated with primary antibodies overnight at 4°C. The membranes were then incubated with relevant secondary antibodies at room temperature for 1 h and visualized by using ECL detection solution (Beyotime, P0018AS). The digital images of the protein bands were acquired using a ChemiDoc MP Imaging System (Bio-rad, ChemiDoc MP). The primary antibodies used in the present study were anti-SIRT7 (*Invitrogen*, PA5-87543), anti-β-actin (CST, 4970L).

### Statistical Analysis

All the presented data and results were confirmed in at least three independent experiments. The data were represented as the mean ± SD and analyzed using GraphPad Prism 7.0 software (San Diego, CA). Statistical significance was analysised by One-way ANOVA, Two-way ANOVA or unpaired Student’s t-tests. ∗ p < 0.05; ∗∗ p < 0.01; ∗∗∗ p < 0.001; ∗∗∗∗ p< 0.0001.

## Data Availability Statement

The original contributions presented in the study are included in the article/**Supplementary Material**. Further inquiries can be directed to the corresponding author.

## Author Contributions

SZ wrote the manuscript. GZ designed experiments. SZ, YL, XZ, MO, GX, and FL performed experiments and analyzed data. ZYW and ZQW provided scientific expertise. LL and GZ supervised the project. All authors contributed to the article and approved the submitted version.

## Funding

This work was supported by the National Natural Science Foundation of China (No. 82170009, 81873958, 82100013), the National Key Research and Development Plan (No. 2020YFA0907200), the Guangdong Scientific and Technological Foundation (No. 2019B1515120041, 2020B1111170014, 2019A1515110055), the Shenzhen Scientific and Technological Foundation (No. KCXFZ202002011007083, JCYJ20180228162511084, JCYJ20190809104205706), and the Sanming Project of Medicine in Shenzhen (No. SZSM201911009).

## Conflict of Interest

The authors declare that the research was conducted in the absence of any commercial or financial relationships that could be construed as a potential conflict of interest.

## Publisher’s Note

All claims expressed in this article are solely those of the authors and do not necessarily represent those of their affiliated organizations, or those of the publisher, the editors and the reviewers. Any product that may be evaluated in this article, or claim that may be made by its manufacturer, is not guaranteed or endorsed by the publisher.
