# Supplementary figures and images for: Sirtuin 7 Regulates Nitric Oxide Production and Apoptosis to Promote Mycobacterial Clearance in Macrophages
Source: Front Immunol. 2021 Dec 3;12:779235. doi: 10.3389/fimmu.2021.779235 (PMC8678072; doi:10.3389/fimmu.2021.779235)

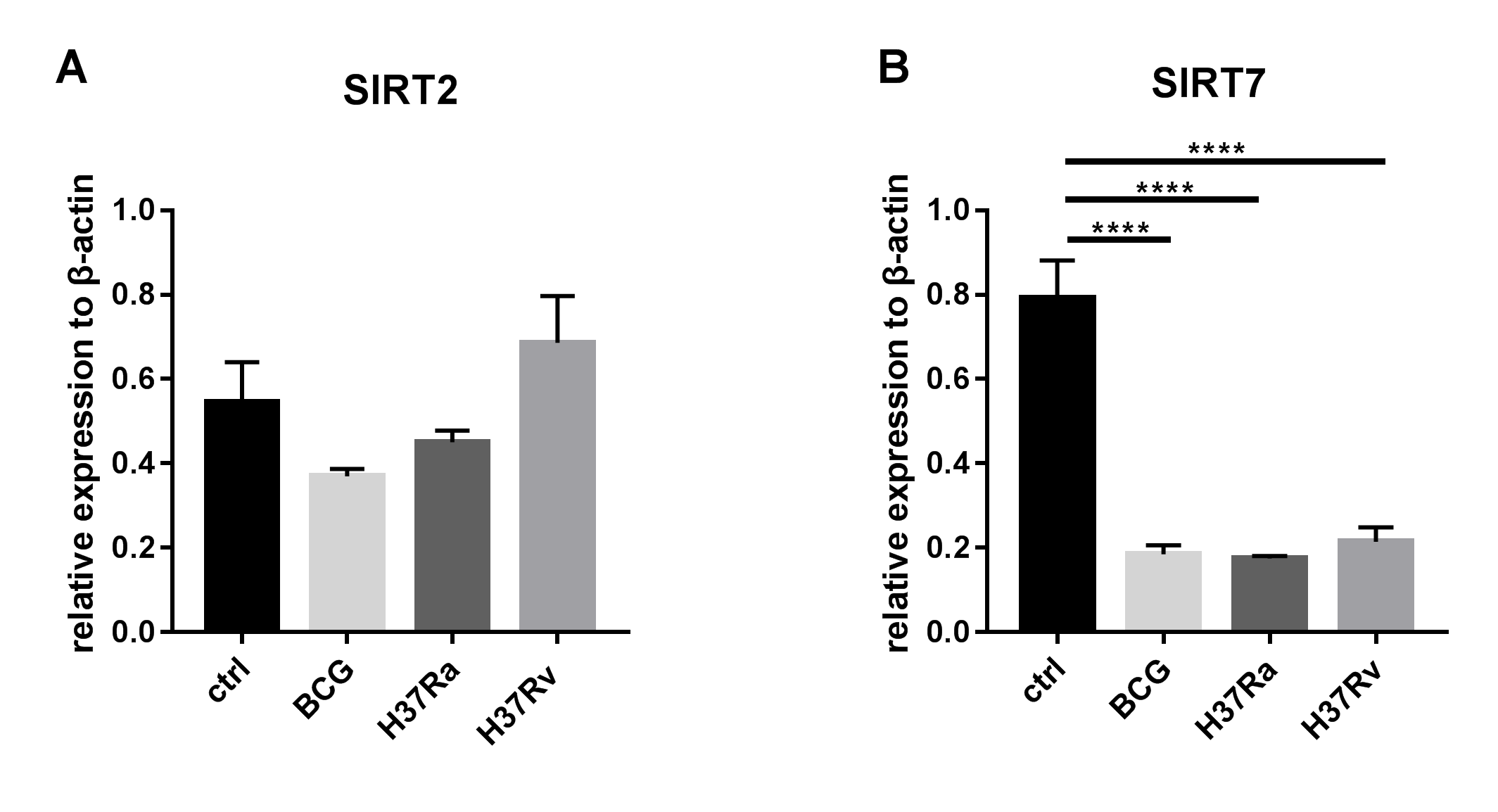

Supplement: Supplementary file 1 [file Image_1.tif]

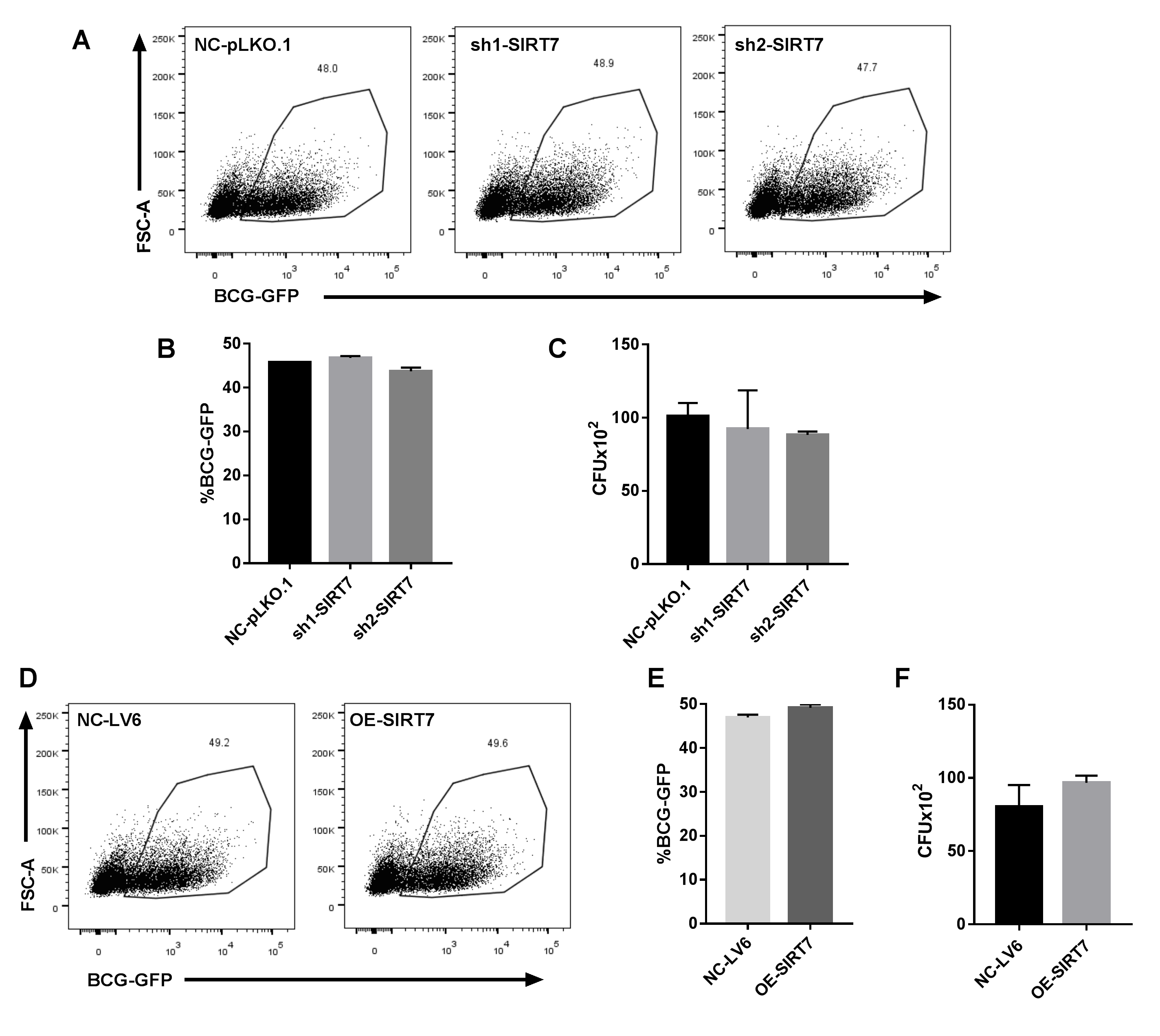

Supplement: Supplementary file 2 [file Image_2.tif]

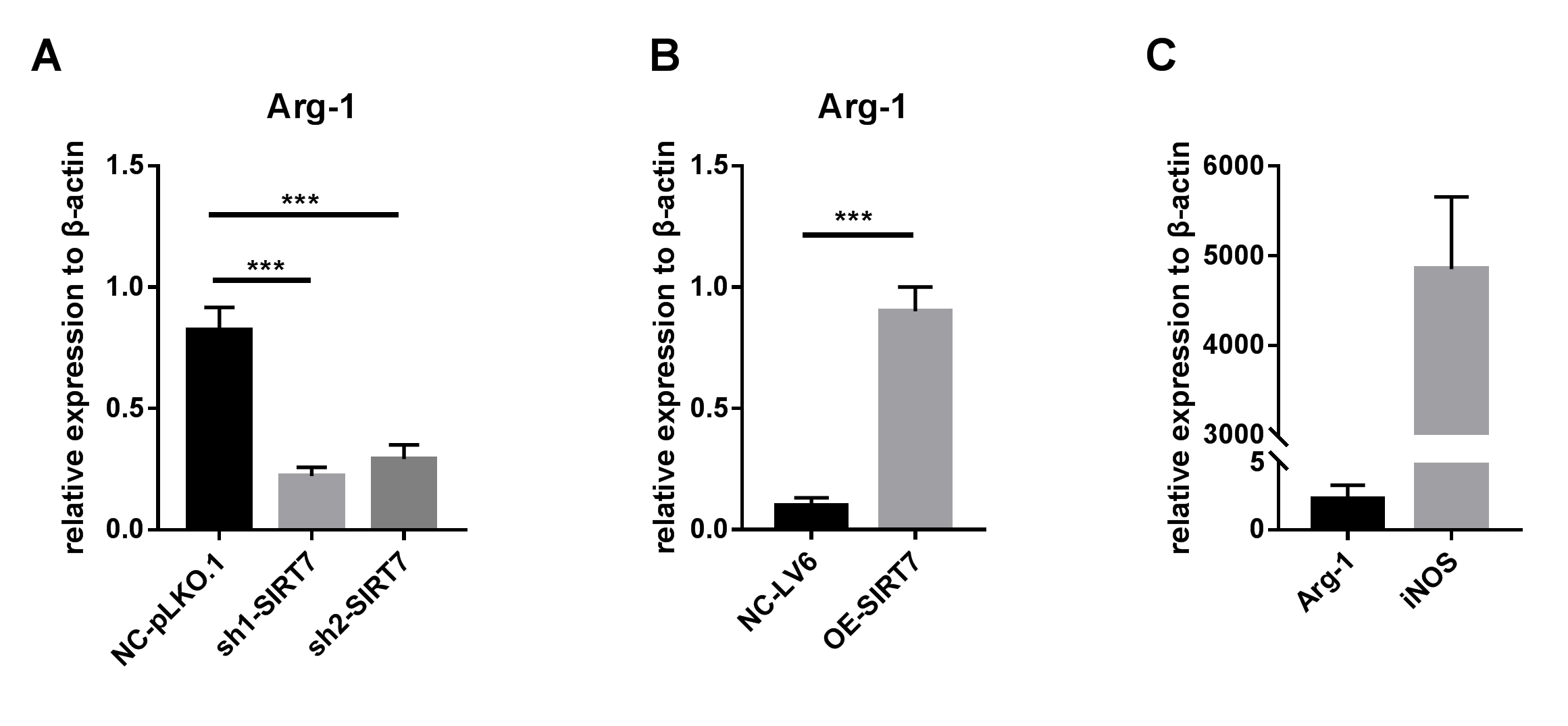

Supplement: Supplementary file 3 [file Image_3.tif]
