# Supplementary material for: Sirtuin 7 Regulates Nitric Oxide Production and Apoptosis to Promote Mycobacterial Clearance in Macrophages
Source: Front Immunol. 2021 Dec 3;12:779235. doi: 10.3389/fimmu.2021.779235 (PMC8678072; doi:10.3389/fimmu.2021.779235)
Supplement: Supplementary file 4 [file Table_1.docx]

**Supplementary Table 1.** The primers used in lentiviral vector construction experiments.

| Primer | Sequence (5’-3’) |
| --- | --- |
| sh1-SIRT7-F | CCGGCTCCATGGGAATATGTATATTCTCGAGAATATACATATTCCCATGGAGTTTTTG |
| sh1-SIRT7-R | AATTCAAAAACTCCATGGGAATATGTATATTCTCGAGAATATACATATTCCCATGGAG |
| sh2-SIRT7-F | CCGGGCAGCTTCTATCCCAGATTATCTCGAGATAATCTGGGATAGAAGCTGCTTTTTG |
| sh2-SIRT7-R | AATTCAAAAAGCAGCTTCTATCCCAGATTATCTCGAGATAATCTGGGATAGAAGCTGC |
| Scramble-F | CCGGGAACGGCATCAAGGTGAACCTCGAGGTTCACCTTGATGCCGTTCTTTTTG |
| Scramble-R | AATTCAAAAAGAACGGCATCAAGGTGAACCTCGAGGTTCACCTTGATGCCGTTC |
| SIRT7-lenti-F | gtcccagactacgcactcgagATGGCAGCCGGTGGCGGT |
| SIRT7-lenti-R | ctgccgttcgacgatggatccCTGCCACTTTCTTCCTTTTTGC |

**Supplementary Table 2.** The primers used in qPCR experiments.

| Primer | Sequence (5’-3’) |
| --- | --- |
| m-SIRT1-F | GCTGACGACTTCGACGACG |
| m-SIRT1-R | TCGGTCAACAGGAGGTTGTCT |
| m-SIRT2-F | CCACGGCACCTTCTACACATC |
| m-SIRT2-R | CACCTGGGAGTTGCTTCTGAG |
| m-SIRT3-F | ATCCCGGACTTCAGATCCCC |
| m-SIRT3-R | CAACATGAAAAAGGGCTTGGG |
| m-SIRT4-F | GTGGAAGAATAAGAATGAGCGGA |
| m-SIRT4-R | GGCACAAATAACCCCGAGG |
| m-SIRT6-F | ATGTCGGTGAATTATGCAGCA |
| m-SIRT6-R | GCTGGAGGACTGCCACATTA |
| m-SIRT7-F | AGCATCACCCGTTTGCATGA |
| m-SIRT7-R | GGCAGTACGCTCAGTCACAT |
| h-SIRT7-F | GACCTGGTAACGGAGCTGC |
| h-SIRT7-R | CGACCAAGTATTTGGCGTTCC |
| m-iNOS-F | GTTCTCAGCCCAACAATACAAGA |
| m-iNOS-R | GTGGACGGGTCGATGTCAC |
| m-ARG-1-F | CTCCAAGCCAAAGTCCTTAGAG |
| m-ARG-1-R | GGAGCTGTCATTAGGGACATCA |
| m-Actin-F | CGCAGCCACTGTCGAGTC |
| m-Actin-R | TCATCCATGGCGAACTGGTG |
| h-Actin-F | ATCATTGCTCCTCCTGAGCG |
| h-Actin-R | CGGACTCGTCATACTCCTGC |

**Figure legends**

**Supplementary Figure 1.** Mtb infection downregulates SIRT7 but not SIRT2 expression in THP-1 cells.**(A, B)** SIRT2 and SIRT7 mRNA expression in RAW264.7 cells infected with Mycobacterial strains with differing virulence. Cells were infected with BCG, H37Ra, and H37Rv, respectively (MOI 10:1) for 4 h, then SIRT2 and SIRT7 expression levels were analyzed 24 h after infection. Data are representative of three independent experiments with similar results and are presented as means±SD. One way ANOVA was performed in **(A, B).** ∗∗∗∗ p< 0.0001.

**Supplementary Figure 2.** SIRT7 knockdown or overexpression has no significant impact on the phagocytosis rate in Mtb infected Raw264.7 cells. **(A, B)** RAW264.7 cells stably expressing scrambled control (NC-pLKO.1) and two independent SIRT7 shRNAs (sh1-SIRT7 and sh2-SIRT7), respectively, were infected with BCG-GFP (MOI 10:1), and then analyzed by flow cytometry at 4h after infection. Representative flow cytometry images of BCG-GFP-positive RAW264.7 cells were captured **(A)** and the percentage of cells positive for GFP were calculated using Flow Jo software **(B)**. **(C)** Colony-forming unit (CFU) counts in scrambled control and SIRT7-knockdown Raw264.7 cells after 4h infecttion with H37Rv. **(D, E)** RAW264.7 cells stably overexpressing SIRT7 (OE-SIRT7) and vector control (NC-LV6) were infected with BCG-GFP (MOI 10:1), and then analyzed by flow cytometry at 4h after infection. Representative flow cytometry images **(D)** and percentage **(E)** of GFP-positive cells were recorded in control and SIRT7-overexpressing cells 4h after infection with BCG-GFP (MOI 10:1). **(F)** CFU counts in vector control and SIRT7-overexpressing cells after 4h infected with H37Rv. Data are representative of three independent experiments with similar results and are presented as means±SD. One way ANOVA was performed in **(B, C),** Unpaired Student’s t-test was used in **(E, F).**

**Supplementary Figure 3.** SIRT7 regulates Arg-1 expression in Mtb infected Raw264.7 cells. **(A, B)** Quantitative RT-PCR analysis of Arg-1 expression levels in **(A)** RAW264.7 cells stably expressing scrambled control (NC-pLKO.1) and two independent SIRT7 shRNAs (sh1-SIRT7 and sh2-SIRT7) or **(B)** cells stably overexpressing SIRT7 (OE-SIRT7) and vector control (NC-LV6) 24 h after infection with H37Rv**. (C)** Relative expression levels between iNOS and Arg-1 in H37Rv infected Raw264.7 cells. Data are representative of three independent experiments with similar results and are presented as means±SD. One way ANOVA was performed in **(A),** Unpaired Student’s t-test was used in **(B).** ∗∗∗ p < 0.001.
